# Critical Hazard Factors in the Risk Assessments of Industrial Robots: Causal Analysis and Case Studies

**DOI:** 10.1016/j.shaw.2021.07.010

**Published:** 2021-07-22

**Authors:** Kangdon Lee, Jaeho Shin, Jae-Yong Lim

**Affiliations:** 1 Department of Safety Engineering, Seoul National University of Science and Technology, 232, Gongneung-ro, Nowon-gu, Seoul, 01811, Republic of Korea; 2 School of Mechanical and Automotive Engineering, Kyungil University, 50, Gamasil-Gil, Hayang-Eup, Gyeongsan-si, Gyeongbuk, 38428, Republic of Korea

**Keywords:** Human factor, Human-robot collaboration, Psychology, Risk assessment, Root cause analysis

## Abstract

**Background:**

With the increasing demand for industrial robots and the “noncontact” trend, it is an appropriate point in time to examine whether risk assessments conducted for robot operations are performed effectively to identify and eliminate the risks of injury or harm to operators. This study discusses why robot accidents resulting in harm to operators occur repetitively despite implementing control measures and proposes corrective actions for risk assessments.

**Methods:**

This study collected 369 operator-injured robot accidents in Korea over the last decade and reconstructed them into the mechanism of injury, work being undertaken, and bodily location of the injury. Then, through the techniques of Systematic Cause Analysis Technique (SCAT) and Root Cause Analysis (RCA), this study analyzed the root and direct causes of robot accidents that had occurred. Causes identified included physical hazards and complex combinations of hazards, such as psychological, organizational, and systematic errors. The requirements of risk assessments regarding robot operations were examined, and three case studies of robot-involved tasks were investigated. The three assessments presented were: camera module processing, electrical discharge machining, and a panel-flipping robot installation.

**Results:**

After conducting RCA and comparing the three assessments, it was found that two-thirds of injury-occurring from robot accidents, causative factors included psychological and personal traits of robot operators. However, there were no evaluations of the identifications of personal aspects in the three assessment cases.

**Conclusion:**

Therefore, it was concluded that personal factors of operators, which had been overlooked in risk assessments so far, need to be included in future risk assessments on robot operations.

## 1 Introduction

Robots have been drawing attention from the manufacturing and service industries, with “unmanned” trends as a core motivation to lead the Industry 4.0 movement [1]. Companies focus on weighing the increasing demand for robots with the numerous operator-injured accidents that mainly occur from operator-robot collisions during robot operations and maintenances.

According to the statistics about operators’ injuries by robot actions from 2009 to 2019, there have been 369 robot-related accident cases reported in Korea in the last decade [2]. Based on the industrial accidents reports from the Ministry of Employment and Labor in Korea (MOEL), the number of robot accidents ranged from 27 to 49 every year, with the highest occurring in 2012 and the lowest in 2007. This is more than twice as many robot accidents as the number from other OECD countries [3].

Currently, a risk assessment is compulsory before implementing robot-related tasks such as assembly, material handling, pick, and place to prevent accidents. This improves safety levels by eliminating and reducing identified hazards. However, despite preventative measures, robot actions are the cause of 30 to 40 accidents per year [3]. In this regard, there are some doubts about whether companies properly perform risk assessments and if there are issues with techniques and evaluations. Relating to these concerns, professionals and various studies emphasize these two points; first, the importance of risk assessment, and second, the consideration of all possible hazards.

According to European Commission guidelines on risk assessment [4], it is recommended that companies identify hazards not only from physical factors but also from psychological, organizational, communicative, cultural, and systematic factors. According to the International Organization for Standardization, all reasonably imaginable hazards that could lead to workers’ injuries should be incorporated into robot risk assessments [5].

Since the introduction of risk assessment guidelines in 1996, there had been noticeable studies about assessments between 1997 and the mid-2000s. In a study on hazardous machinery [6], a risk assessment checklist was designed, and only mechanical and electrical items, the state of deterioration, and managerial aspects were included in the checklist. For machine tools and metal-related companies, the status of a risk assessment was surveyed, and only visible risks were included in the assessment [7].

After the mid-2000s, many professionals started to be concerned about aspects of risk assessment other than physical factors. One study [8], dealing with the risk assessment of machines, investigated a few risk theories to reflect the psychological, cultural, and communicative perspectives; it pointed out that risk assessment is a crucial tool that enables employees to use machinery safely and make risk-informed decisions. In addition, Russ et al. [9] demonstrated that human and environmental factors were the leading causes of industrial machinery accidents in the UK in 2009.

For robot risk assessments, it was pointed out that the physical capabilities of the robot, work environment, and operators were not considered when conducting a risk assessment of industrial machinery [10]. Likewise, a case study [11] has not fully evaluated the items recommended by the EU and ISO when the hazards of robots, especially operating closely to workers without appropriate barriers, were investigated. Only physical hazards were included, but work environments, human aspects, and organizational climates were not.

The above-mentioned literature on risk assessments of industrial robots has thus far raised the crucial issue that most risk assessments evaluated only the physical hazards and visible risks, without including other factors such as operator, job, and system aspects recommended by international standards. There have been no studies about the unconsidered and omitted evaluation items and about which methodologies and criteria need to be added to currently conducted assessments in practice. Furthermore, risk assessments of machining tools and cranes merely evaluated apparent hazards, and only a few risk assessment cases focused on evaluating hazards other than physical aspects [12,13].

In this regard, the purpose of this study is to discover the unconsidered and omitted items from risk assessments of industrial robots and to suggest evaluation factors and detailed classifications be added. This article is organized as follows: In Section 2 (Materials and Methods), operator-injured accidents during the last decade are categorized based on accident classification systems, and the three cases of robot risk assessments in practice are also presented in this section. The following section, Section 3 (Results), analyzes root causes of robot accidents after investigating direct causes. The latter part of Section 3 demonstrates that most risk assessments in use have overlooked the significance of operator, job, and system-related risk factors and suggests some critical risk factors to be considered. Section 4 (Discussions) briefly discusses a few of the research limitations. The last section, Section 5 (Conclusion), provides future directions regarding risk assessments on robot operations.

## 2 Materials and methods

To examine specific risk factors on workers when in robot operation, the following two kinds of jobs are included: analysis of previous operator-injured accidents and analysis of risk assessments in practice. The former is about types, frequency, and injury significance in previous robot accidents. The latter is an analysis of risk assessment cases of robot operations in practice. This section demonstrates the accident analysis of industrial robots during the last decade and the three risk assessment cases.

### 2.1 Operator-injured accident cases by industrial robots

This subsection presents the types of robot accidents and work processes in which the robot accidents occurred and the corresponding lost working days of injured operators. These cases of robot accidents had been collected from the statistics book of industrial accidents issued by the Ministry of Employment and Labor (MOEL) in Korea [2] and accidents investigation reports between 2009 and 2019 derived from the Korea Occupational Safety and Health Agency (KOSHA).

We have examined diverse statistics data from the MOEL website, which updates statistical information about industrial accidents and injury-related data every month, quarterly, and once a year. Moreover, this study has collected accidents investigation reports from safety material repositories of the KOSHA website and has revisited robot-concerned published research reports to adopt meaningful statistics data from occupational safety and health research institute (OSHRI) in KOSHA.

The robot accident information consists of descriptions of accidents, direct causes, and other accident-related elements. For example, descriptions of accidents explain how they occurred with information on time, place, people, work, and process. Direct causes of robot accidents are immediate reasons for operator-injured accidents that initiate wrong behaviors or undesired events. Root causes are fundamental agents that result in direct causes and can be discovered by analyzing accident reconstruction and direct causes [14,15].

This information is categorized as types of accidents and works, and operators’ injured parts. Causes of accidents are classified into both direct reasons with unsafe behaviors and conditions and root causes from person-, job-, and system-related factors with the Systematic Causal Analysis Technique (SCAT) that was developed by Det Norske Veritas (DNV) [16] with original ideas from James Reason’s Swiss Cheese Model [17]. SCAT has predominant popularities in various business settings, including industrial sectors, as one of the root cause analysis methods.

### 2.2 Case studies on risk assessments of industrial robots

For a better interpretation of case studies on risk assessments of industrial robots, understanding the principles and procedures of risk assessments is required. A risk assessment is a repetitive task that every workplace should follow to maintain safety at a reasonably practical level by identifying, estimating, evaluating every potential hazard, and reducing the risks as followed [5]. Risk assessments are based on the principles of safety of machinery from ISO 12100. Most companies in Korea perform risk assessments by utilizing the 4M method supported by KOSHA, which assesses the risks from the aspects of Man, Machine, Media, and Management [18].

For selecting appropriate risk assessment cases on industrial robots, this study interviewed three subject matter experts (SMEs) from major electronic parts manufacturing businesses in Korea with more than 1,000 workers. During the interviews, SMEs were asked about what types of critical risks exist in working with industrial robots, how risk assessments should be performed on robots, and which job or operation should be analyzed in detail, reflecting its riskiness.

In addition, we have examined several risk assessments of industrial robots at three above-mentioned enterprises, both in documents and on the spot with SMEs. And this study has decided to adopt three risk assessments as materials for case studies after looking into their documents of assessing risks on robot operations, operators’ job performances, and interviews from some of the workers at those sites.

The three risk assessments in subsection 2.1 for job processes using industrial robots: camera module processing for smartphones; electrical discharge machining for mold manufacturing; and recovery of a panel-flipping robot are scrutinized. For methods of risk identifications and evaluations, ISO 12100 for machine safety [5] and 4M (Man, Machine, Media, Management) assessment tools [18] are utilized in the three cases relating to assessment scope, method, and items by revisiting the cases.

#### 2.2.1 Case I: camera module processing for smartphones

**Fig. 1:**
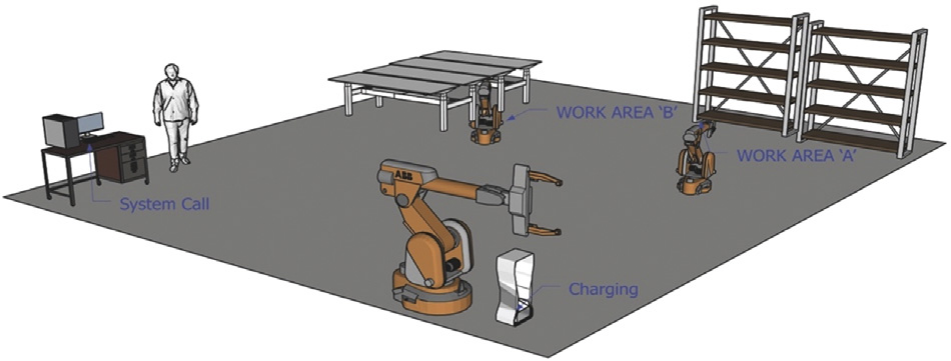
Camera module processing for smartphones.

The following sequence completes the work process: system call, robot moving, module loading, assembling and module unloading, robot moving back, and system stop, as illustrated in Fig. 1. In Case I, human-robot interactions occur both when an operator loads camera modules on the loading station (work area A) and when an operator unloads sockets with camera modules after a robot finishes assembly jobs (work area B). There are no physical barriers between an operator and a robot, although the distance between the two is less than 2m during the interaction. An operator wears personal protective equipment such as a safety helmet, a safety goggle, and earplugs.

[work procedure] System call → Module loading at the work area A (Human-Robot Interaction) → Assembling at the work area B → Socket with module unloading at B (Human-Robot Interaction) → Robot moving back at A → System stop.

For this robot-related work process, risk assessment was implemented by the following steps. First, the company identified hazards by applying the criteria specified in ISO 12100 [5] to a camera module processing line. Next, the risk estimation was performed using the risk graph method referenced from ISO/TR 14121–2 [19]. The assessments found 27 hazards in total: 21 mechanical, four electrical, and two ergonomic. Moreover, 20 out of 27 hazards needed immediate risk reduction measures, such as proper personal protective equipment (PPEs). The others did not require further protective measures due to low risk.

#### 2.2.2 Case II: electrical discharge machining (EDM) for mold cores

**Fig. 2:**
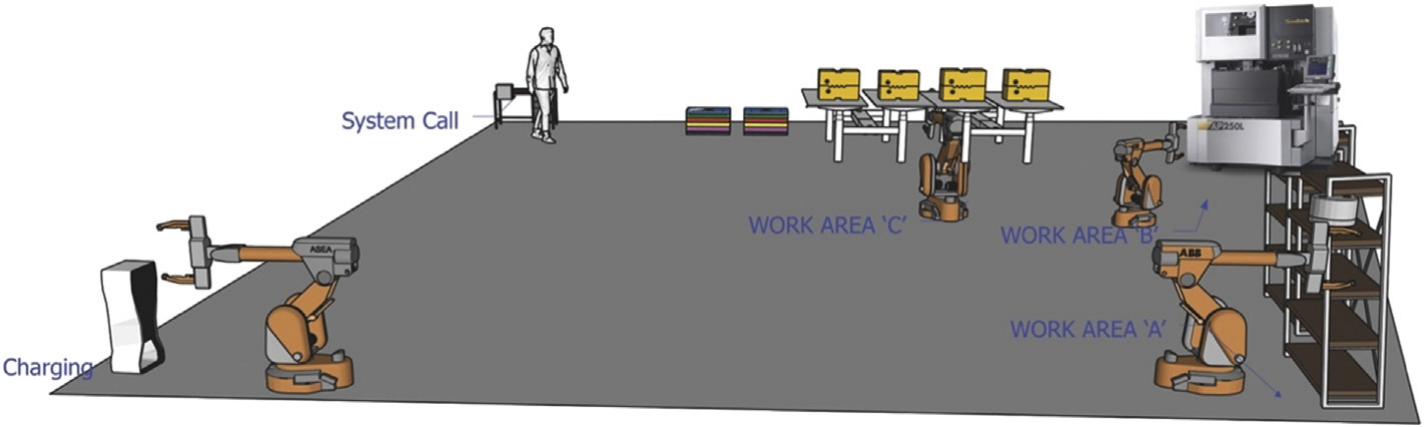
EDM work process for mold core.

This work was accomplished by the sequence of moving and locating mold cores, electrical discharging, cleaning, moving back to the original position, and stopping the system, as shown in Fig. 2. In Case II, human-robot interactions occur both when an operator loads mold cores on the loading station (work area A) and when an operator unloads mold cores after finishing electrical discharging jobs (work area C). There are no physical barriers between an operator and a robot, although the distance between the two is less than 1.5 m during the interaction. An operator wears a safety helmet, a safety goggle, earplugs as personal protective equipment while working closely with a robot.

[Work procedure] System call → Moving and locating cores at the work area A (Human-Robot Interaction) → Electrical discharge machining at the work area B → Cleansing and cleaning at the work area C (Human-Robot Interaction) → Robot moving back → System stop.

The 4M method developed by the Korea Occupational Safety and Health Agency [18] was used for the risk assessment. Ten hazards were identified, and upon reclassification, three were in the mechanical category, one in chemical, two in ergonomic, and four in managerial.

#### 2.2.3 Case III: dismantling and recovering of a panel-flipping robot

**Fig. 3:**
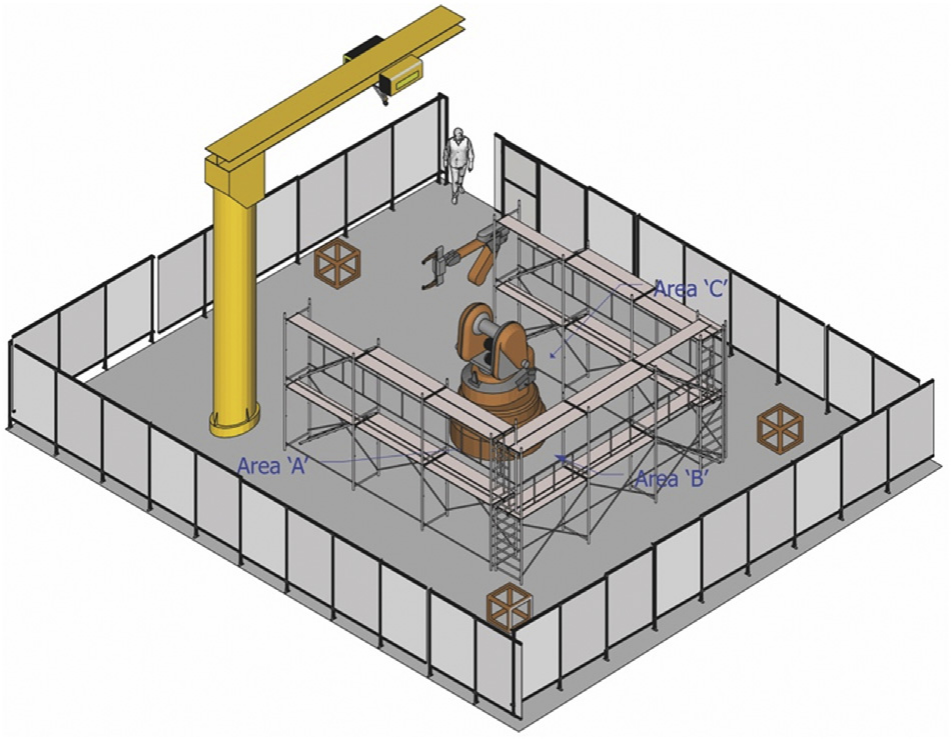
Dismantling and recovering of a panel-flipping robot.

This work was turning and flipping a glass panel upside down by a robot operation and consisted of moving and docking, installing a robot and cables, adjusting, and testing, as shown in Fig. 3. In Case III, human-robot interaction occurs only when an operator takes a test working and inspects a robot’s movement with hand guiding and micromotion after completing the installation of a robot. Unlike Case I and II, there are physical barriers between an operator and a robot, but an operator works inside the physical barriers equipped with a safety helmet, a safety goggle, a safety harness, and earplugs for one’s protection during installation and inspection.

[work procedure] Moving and docking → Robot installation (A) → Installing cables and pipelines (B) → Robot locating and adjusting (C) → Robot testing (Human-Robot Interaction) → System start.

The risk assessment for this work was also performed using the 4M method [18]. The total risks identified were 33; 22 in mechanical, one in electrical, seven in ergonomic, and three in work environment hazards.

## 3 Results

### 3.1 Analysis of operator-injured robot accidents in Korea during the last decade

#### 3.1.1 The current state of robot accidents and direct causes

From the last ten years (2009–2019), there have been 369 operator-injured accidents by robot-related tasks reported in Korea. In this study, the direct causes of all reported accidents have been examined; then, the root causes resulting in the direct causes have been traced based on cardinal information about the accidents: overview, type of accident, part of the injury, work procedure, and so forth [2].

According to the statistical data, the number of yearly robot accidents ranged from 27 to 49, the highest occurring in 2012 and the lowest in 2007. This is consistent with a similar investigation [3], which states there are 30 to 40 accidents every year on an average—more than twice the number of robot accidents in OECD countries.

**Fig. 4:**
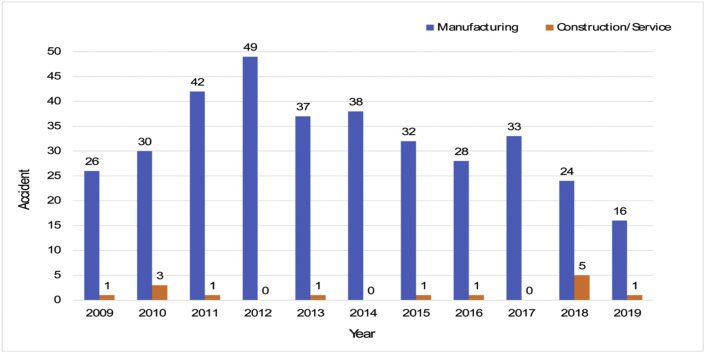
Annual robot accidents by industry (2009~2019).

More specifically, Fig. 4 shows that more than 95% of the robot-related accidents— 355 cases—occurred in manufacturing businesses, while the remaining 14 were reported from the service and construction sectors. The primary reason for the high percentage of accident cases in manufacturing settings corresponds to the statistical fact that manufacturing businesses utilize 89% of industrial robots in total, as compared to 11% of service industries’ utilization [20].

**Fig. 5:**
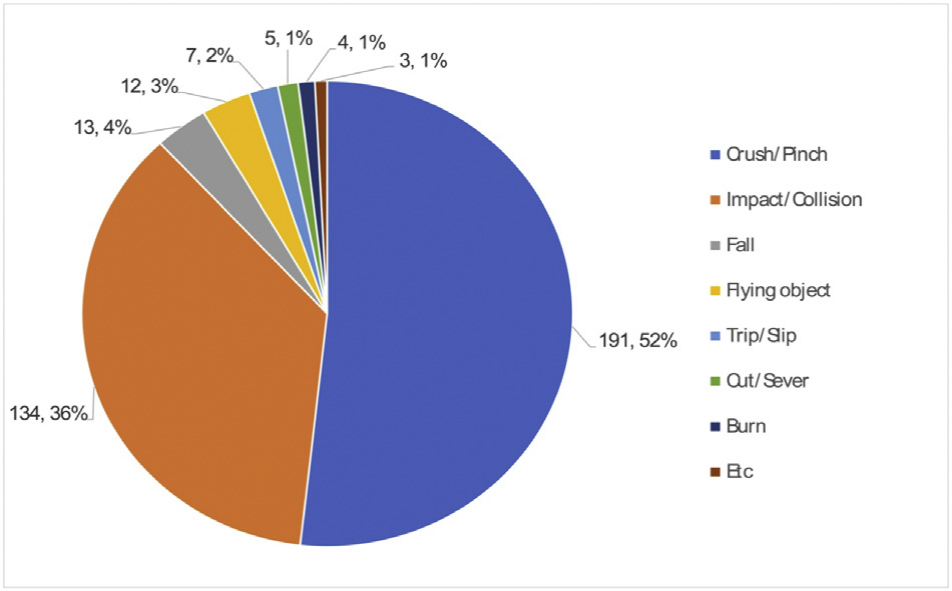
Types of accidents by industrial robots (2009~2019).

The 369 robot-related accidents are classified by accident type, as illustrated in Fig. 5. Two types were predominantly responsible for 325 (88%) of the 369 accident cases: the first type, with 191 (52%) cases, corresponded to “jammed,” “caught in,” or “crushed” cases, and the second type, with 134 (36%) of the cases, corresponded to “collision” or “impact” cases. The first type occurred when a part of the body was stuck either between a fixed structure and robot arms or between fixed and moving parts. It also occurred when a body part was placed between more than two moving parts of industrial robots. In the “collision” or “impact” cases, most of them related to workers’ irresponsible approaches to the robot or the sudden start-up of the robot when closely working near the machinery sites.

In addition, “falling” from a height during maintenance and “hit by” or “struck by” a flying object caused by the separation of robot parts or tools are the next most common robot-related accidents. The two types consist of 13 (4%) and 12 (3%) of the total types, respectively. The remaining types include accidents caused due to “slipping,” “tripping,” “amputation,” and “burn.” This analysis is slightly different from the previous investigation on industrial robot-related accidents from 2011 to 2015 in Korea [3], in that “hit by” or “struck by” was the third most common type of accident, consisting of 5% of the cases, and “falling” was the fourth type, with 4% of the total accidents in that period.

**Fig. 6:**
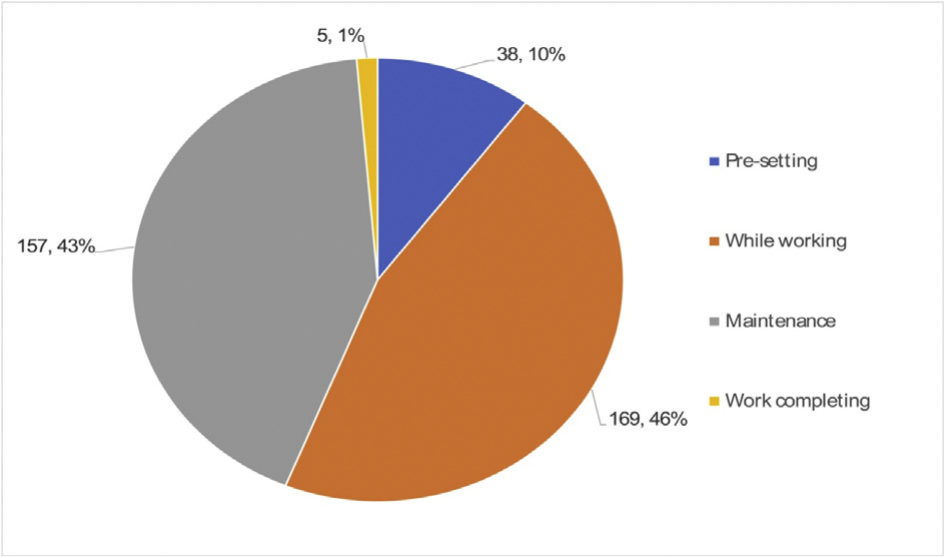
Types of works in which accidents occur.

The next investigation was on the relationship between job tasks and accident possibilities. In other words, we explored the task that was closely related to accidents. As shown in Fig. 6, 46% of accidents occur when they are in operation. However, these are involved in abnormal jobs. The three representative jobs in operation are enumerated as follows: taking off a foreign substance during operation without stopping it properly; observing whether products are in good order, or a sudden operation of the robot while taking emergency measures.

The number of accidents that occurred in maintenance is comparable to the ones that occurred “while working,” as it takes up 43% of 369 cases. In the rest of the cases of robot accidents, 11% of the cases occurred in the installation or the preparation of the robot. In short, most robot-related accidents are associated with robot operation and maintenance. Similarly, this result is in line with previous investigations on 273 robot-related accidents between 2011 and 2015 [3].

**Fig. 7:**
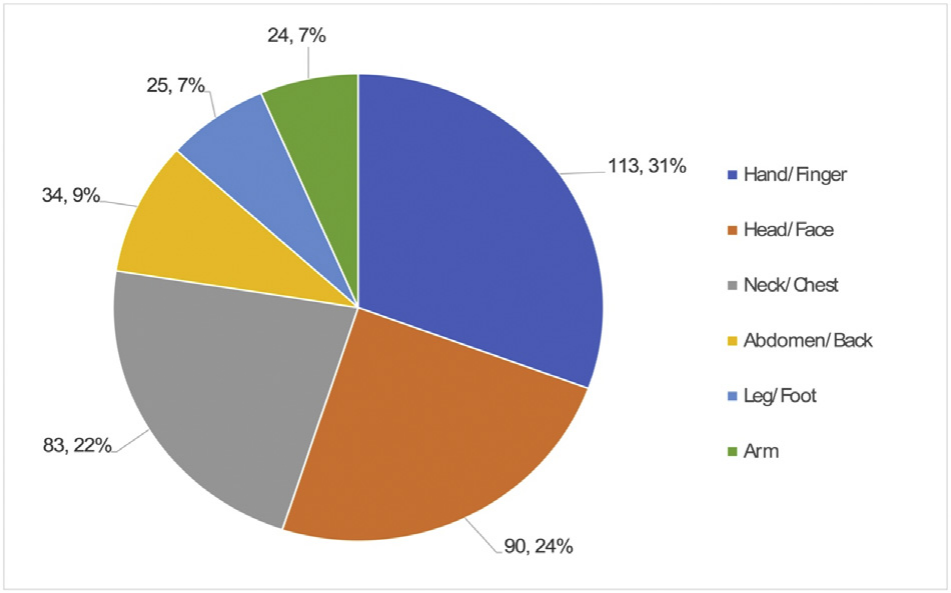
Damaged parts of the human body caused by industrial robots’ accidents.

The following investigation was concerned with injured body parts to understand the parts most exposed to hazards. Fig. 7 shows the body parts that are most susceptible to injury when they meet with an accident. Hands and fingers are the most vulnerable, as seen in 113 cases (31%), followed by head/face and neck/shoulder/chest, amounting to 90 cases (24%) and 83 cases (22%), respectively. Other parts, such as the abdomen and back, legs, and arms are also among the affected ones. Additionally, apart from operators’ external injuries by robots, chronic injuries within robotic work environments such as noise-induced hearing loss, skin hypersensitivity, white knuckle due to vibration, etc. need to be analyzed as well. However, this study has focused more on traumatic injuries than on cumulative effects that are difficult to obtain and analyze the data about chronic-related injuries.

#### 3.1.2 The root cause analysis (RCA) of robot accidents at work

As stated in Section 3.1, there were 34 cases of robot-related accidents every year, most of which were associated with manufacturing. The majority of accidents occurred due to “crushing” and “collisions” during abnormal operations and maintenance jobs.

However, it is more crucial and effective to eradicate the causes of operator-injured accidents by robots rather than to develop short-term strategies or reactive measures for the direct causes. It was also emphasized that system safety, including design, space, and repair, is necessary to prevent robot accidents [21,22].

Since fixing direct causes cannot be the primary measure to reduce the number of robot accidents, it is worth utilizing root cause analysis to investigate the origin of the direct causes [17].

RCA is an approach to find and analyze the origin of an accident [23]. A loss causation model [15], known as the New Domino Theory by Frank E. Bird, was adopted to analyze robot accidents in this study, along with the systematic cause analysis technique (SCAT) [16].

For eradicating repetitive accidents in industrial settings, it is more valuable to find root causes from personal and organizational factors than to look for direct causes to fix undesirable behaviors and conditions as quickly and easily as possible [14]. For example, SCAT categorizes the root causes of accidents or losses into two groups: personal factors and job and system factors. Personal factors include inadequate physical or mental capability, and job and system factors contain organization, leadership, work standards, communication, psychology, and behavior.

**Fig. 8:**
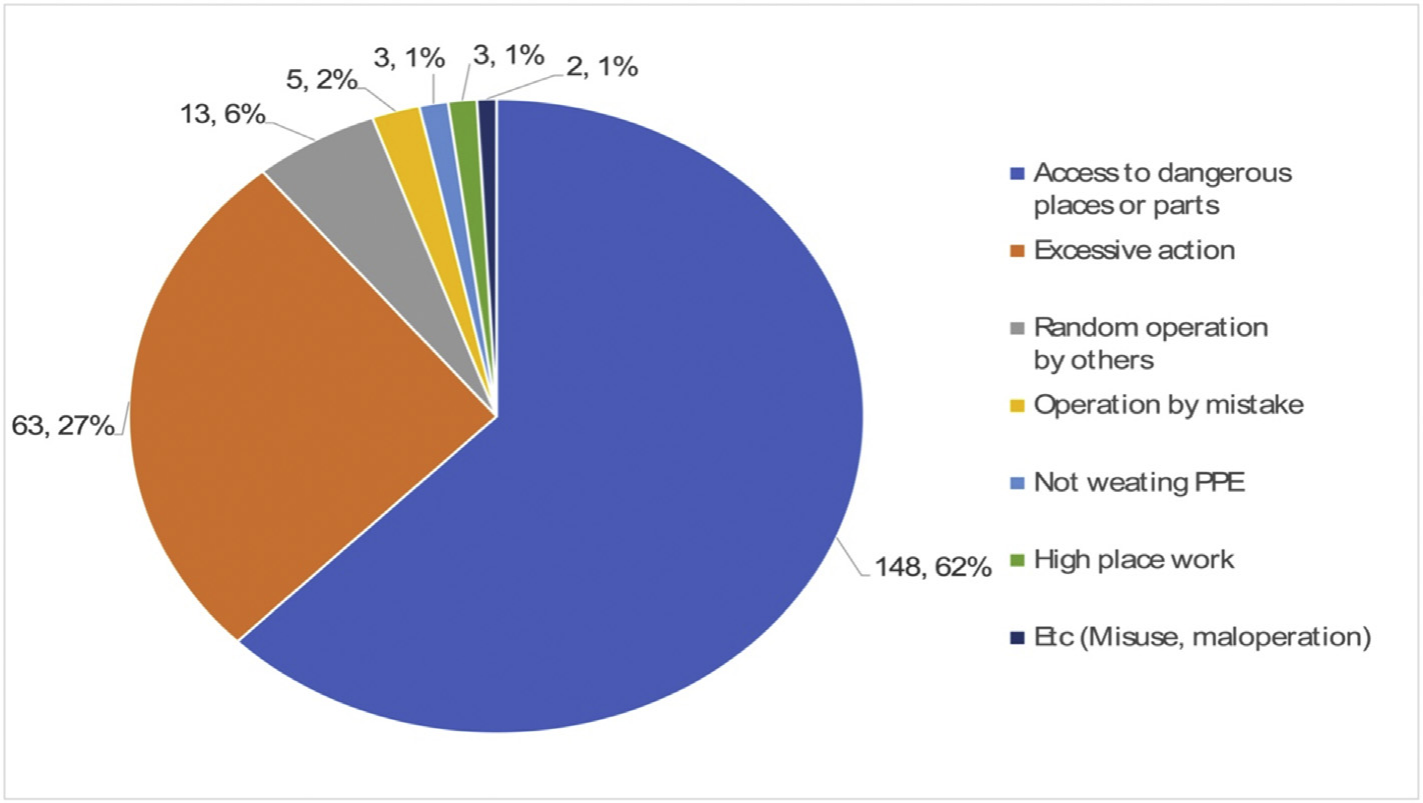
Direct cause (Unsafe behavior, 237 cases).

The direct causes of robot accidents analyzed in Section 3.1 are classified into two types: unsafe behavior and unsafe conditions. As shown in Fig. 8, the direct cause of 237 out of 369 accidents was unsafe behaviors. Among the immediate causes of the 237 accidents, “access to dangerous places or parts,” “excessive action or movement,” and “random operation by others amounted to 148, 63, and 13 cases, respectively.

**Fig. 9:**
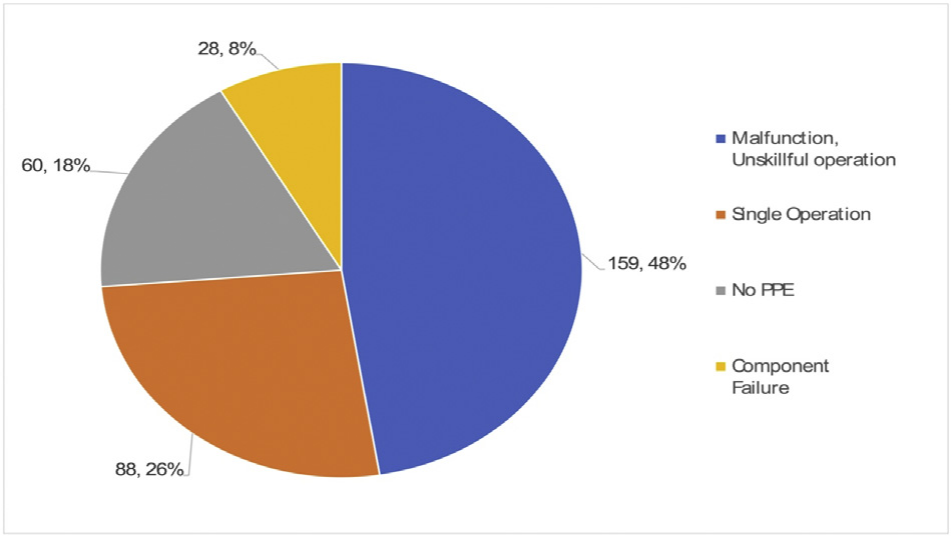
Root cause (Improper physical capability, 335 cases).

**Fig. 10:**
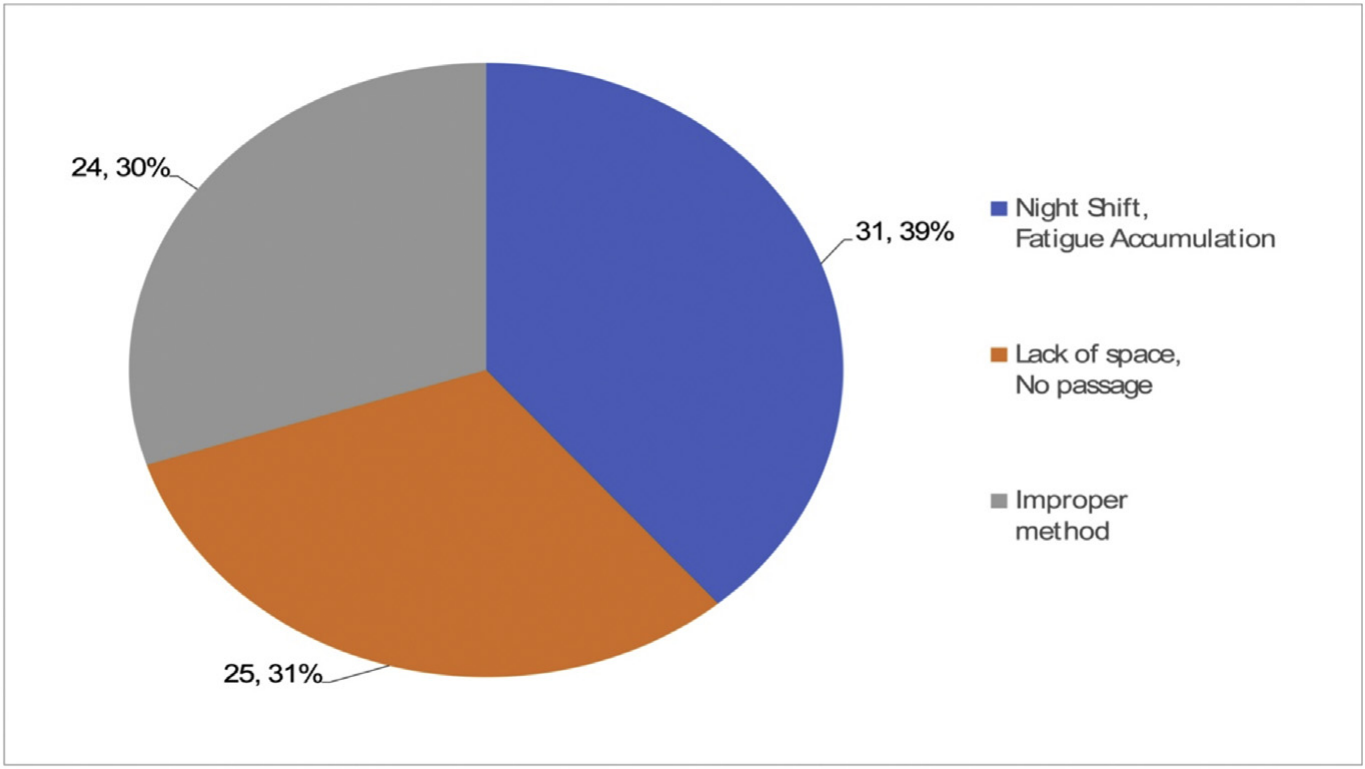
Root cause (Improper physical stress, 80 cases).

“Access to dangerous places or parts” at work is thought to be caused by psychological pressures and stress such as heavy workload, haste, inappropriate work order, and a tight delivery deadline. “Excessive action or movement” can be caused due to a lack of workforce, night shifts, shortage of relaxation, or fatigue. “Random operation by others” can be the result of a lack of job- and system-related management, such as vague job descriptions, no working procedures, absence of communication, no installation of safety devices, and poor planning. The root causes for unsafe behaviors and improper jobs can be attributed to personal factors like lack of physical/mental capability or physical/psychological stress, as shown in Fig. 9, Fig. 10.

**Fig. 11:**
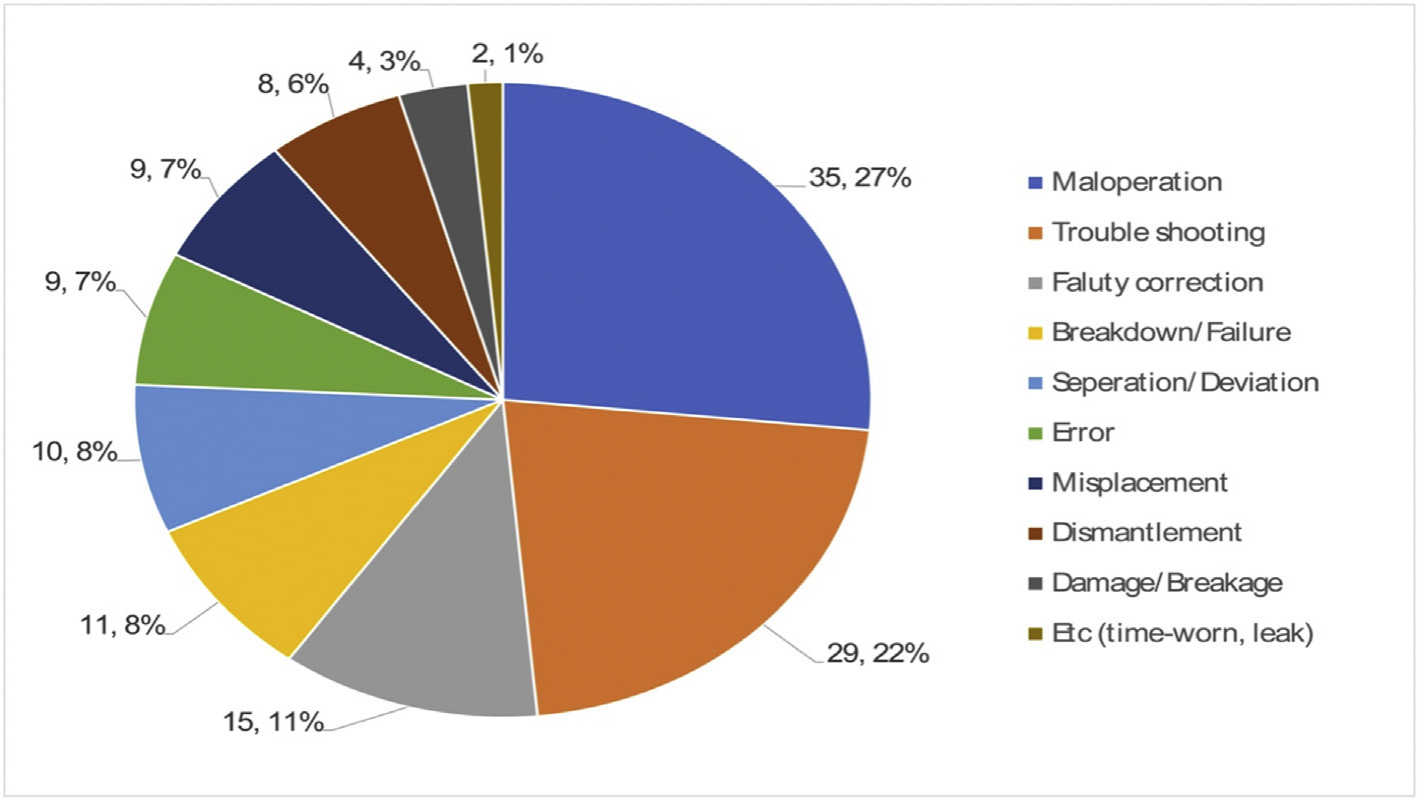
Direct cause (Unsafe condition, 132 cases).

On the other hand, the remaining 132 out of 369 robot accident cases can be said to be caused by unsafe conditions. These can be categorized into three main causes: “maloperation,” “troubleshooting,” “faulty correction and breakdown/failure,” which, as shown in Fig. 11, amounted to 35, 29, and 26 cases, respectively.

**Fig. 12:**
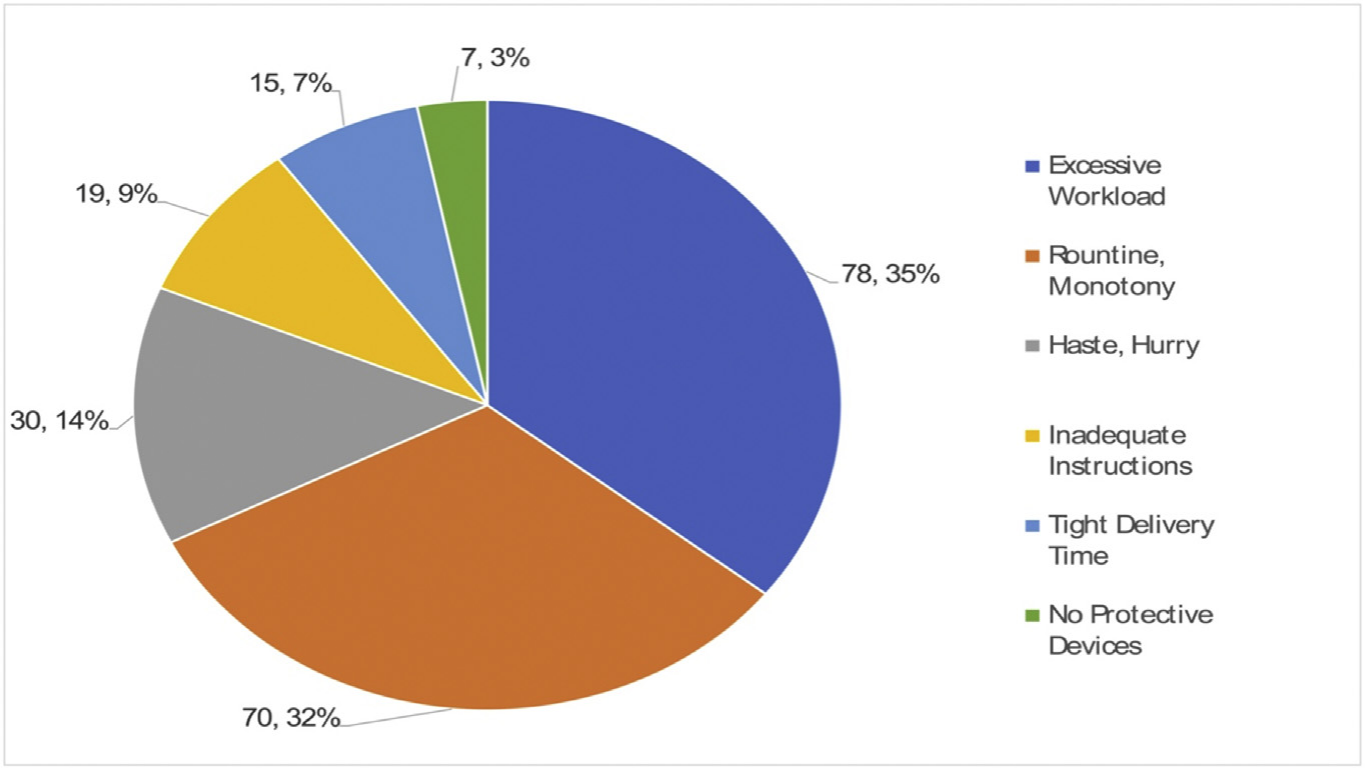
Root cause (Improper mental stress, 219 cases).

**Fig. 13:**
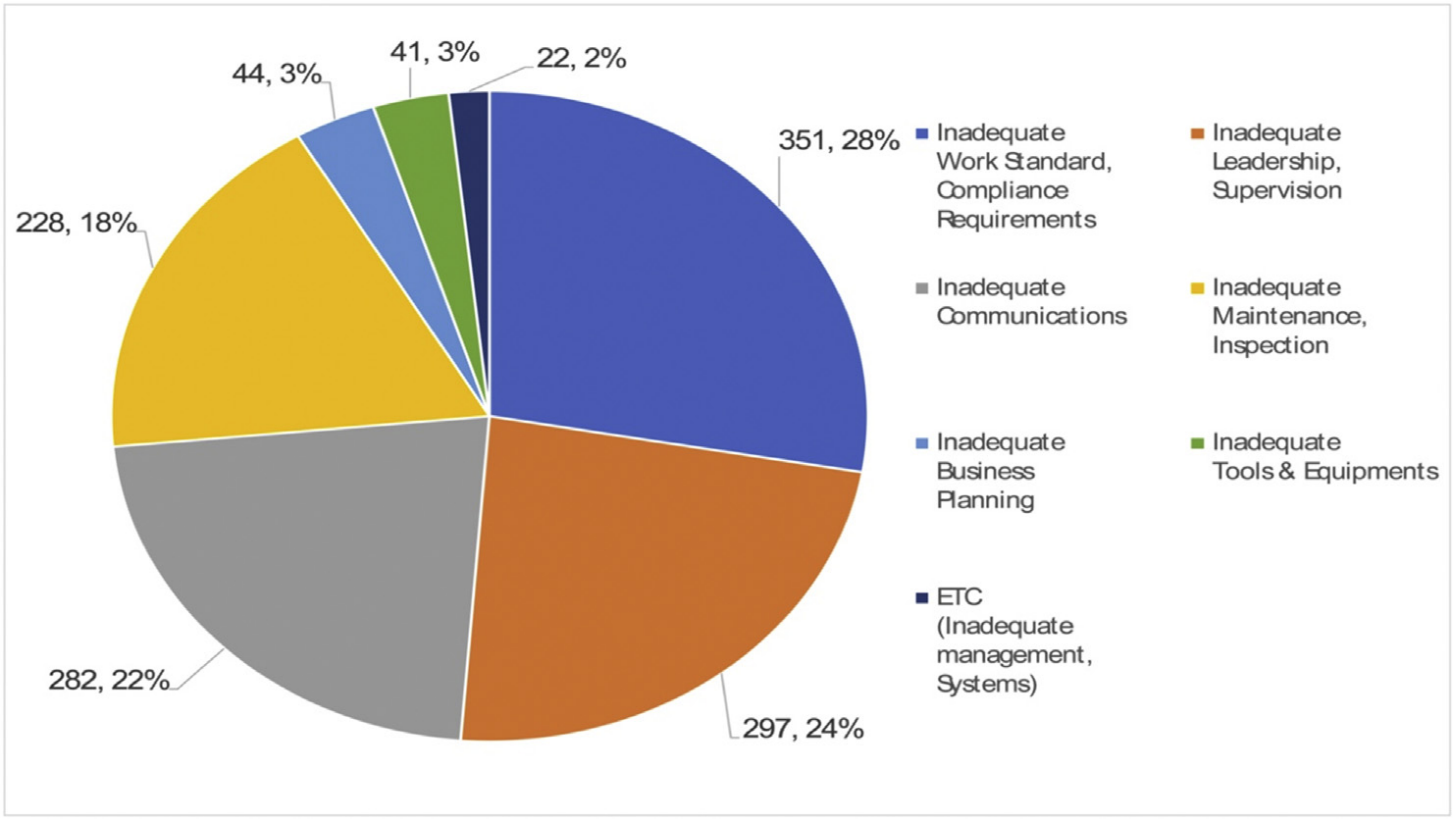
Root cause (Job & System, out of 369 cases).

Some reasons for why unsafe conditions still remained until an accident occurred can be found in personal factors, such as inappropriate physical capability and psychological stress, and job and system factors, as seen in Fig. 12, Fig. 13.

For example, the reasons for “maloperation by workers or malfunction of robots” can be found in narrow workspace and wrong layout, and disobediences of accurate operation time by working alone. “Error correction without complete stop” occurs in routinely repetitive jobs and under forced circumstances of complying with tight deadlines, which makes workers irritated and impulsive. The accidents that occur in “correcting breakdown” are associated with utilizing assembly parts with low reliability, lack of supply and demand for components, and no work standards and approval procedures.

Given the RCA analysis in section 3.2 on 369 robot-related accidents from the last decade, it can be seen that the root causes leading to direct causes can be categorized into personal and job/system factors; it also shows that the risk assessment cases of industrial robots presented in section 2.1 contain neither personal factors nor job/system factors.

However, despite the significance of the above-mentioned personal and job/system factors, they have been overlooked in the previous risk assessments of industrial machinery such as tower cranes, forklifts, and injection molding machines, etc. [12,13,[24], [25], [26], [27]].

For this reason, we carefully claim that the occurrence of 30–40 robot-related accidents every year is attributed to many omissions and misevaluations of risk assessment items. To put it another way, when performing risk assessments on industrial robots, companies, while assessing the risk at their working sites, should include not only physical, tangible factors but also nonphysical factors such as personal, job, and system factors, which are recommended by ISO 12100 [5] and EU Directive 89/391/EEC [4].

### 3.2 Analysis of case studies for robot risk assessments

The international standard of risk assessments on industrial machineries was originated from the EU’s safety and health framework at the workplace, Directive 89/391/EEC [4], enacted in 1989 and was spread by ISO 12100 [5], the safety of machinery: general principles, in 2010. Enterprises have been performing risk assessments based on EU and ISO criteria, repetitively implementing procedures from hazard identification to risk evaluation. In these two criteria, there are ten to eleven evaluation items, including physical and external risk factors, to identify, estimate, and evaluate the risks. Some of the detailed items to evaluate are listed below.

- EU: equipment, work practices, use of electricity, exposure to substances, environmental factors, working climate, psychological factors, work organization, etc.
- ISO: mechanical, electrical, thermal, noise, material, ergonomic, environmental, etc.

In accordance with the international standard of risk assessments on industrial machinery explained in subsection 2.2, a risk assessment needs to thoroughly evaluate not only physical and visible risks but also worker, job, and system-related risks. However, the three presented risk assessment cases have something in common; All of the three cases considered only some physical and ergonomic hazards and exclude worker factors (psychology and job stress), job factors (work organization and difficulties of tasks), and system-related factors (alarm system, cybersecurity, etc.).

From the previous three risk assessment cases, two points can be noticed. The first is regarding the limited hazard types at the identification stage. The companies identified only externally observable hazards in the three cases, i.e., those which are visible and tangible. However, the list of identified hazards does not include all the hazards leading to accidents. In other words, accident-inducing hazards to robot operators include psychological, organizational, communicative, cultural, and security factors. Moreover, ISO and EU recommendations consistently consider these above-mentioned hazard factors as significant.

The second point is the omission or misevaluation of observable hazards from operators’ behavioral aspects and working conditions. It appears that the hazards resulting from workers’ behaviors and the conditions of machines were considered negligible when performing the risk assessment. In the previous cases, potential hazards harmful to workers might not have been included due to omitting or misevaluating hazards. Thus, externally perceptible hazards should also be scrupulously identified during the risk assessment.

### 3.3 Improvements in the items of operator-injured accident causation and risk assessment

By comparing operator-injured accidents with three risk assessments of robot operations, there have been some gaps between causes of accidents and results of assessments. In other words, many accident-induced causes have not been considered properly or been omitted in robot risk assessments. In accordance with the current risk assessment practices of industrial robots, two weaknesses were discovered in terms of risk finding and accident causation.

The first weakness is that many evaluation items are likely to be neglected in the risk assessment of robots without considering comprehensive hazard factors that are recommended by ISO 12100 and EU Directives. The second is that the estimated items are only determined by assessing physically exposed hazards. Even observable risks are misevaluated without considering the root causes of robot accidents.

There are 11 evaluation items of risk assessment within four categories in the EU Directive [4] and ISO 12100 [5] to comply with and apply to the work sites.

By judging from the three risk assessment cases of industrial robots in this study, there seems to be only a number of physical items, such as mechanical, electrical, chemical, and hygienic, to identify and estimate hazards during risk assessments. These partial and inadequate identifications and evaluations of hazards might lead to similar accidents by robots.

However, as consistently emphasized, companies should identify and evaluate hazards not only from the physically visible aspects but also from personal, behavioral, task-based, and system-related hazards, as reasonably foreseeable as possible. Moreover, root causes, together with direct causes, need to be identified to minimize robot-related accidents. The applications of RCA methodologies such as “SCAT,” “TapRoot,” “Apollo,” “Tripod,” and “Five Whys” are recommended for accident reconstruction and root cause mapping [14,15].

## 4 Discussion

In this study, unconsidered but critical hazard factors in the root causes of operator-injured robot accidents and risk assessments of robot operations have been scrutinized by analyzing the accident cases of robots for the last decade and three cases of robot risk assessments. This means that only physical and visible hazards in present risk assessments on industrial robots have been evaluated. Therefore, this has led to the results pointing out that robot risk assessments should contain hazard factors related to humans, jobs, and systems more closely in order to prevent similar accidents by robots.

However, this study has a number of limitations despite the above-mentioned results. Firstly, this paper needs to be concerned about work environments from diverse industries because three cases of robot risk assessments are derived from some of the electronic component manufacturing companies. Moreover, the correlation of accident causal factors such as type of employment, age, sex, job position, career, and so on have not been identified, although this study analyzes the root causes of operator-injured robot accidents.

Furthermore, besides operator-injured accidents by robot movements, chronic injuries such as hearing loss, vibration, and exposure to hazardous material have not been considered because of difficulties in obtaining and analyzing chronic-related data and the long latency period of chronic diseases. The last limitation of this study is that further assessment scope, item, and degree of robot-related significant hazards demonstrated in Table 1 have still remained undiscovered for future research, although the result of this study suggests other critical hazard factors rather than physical elements be considered to improve human-robot trust for safe work environments within industrial robots.

**Table 1:** Classification on the hazard category of risk assessments on robots [4,5]

Category (factor)		Detailed classification	Remarks
1. Physical	Mechanical	Use of work equipment, work procedure and method, layout of premises, etc.	As-Is
	Electrical	Use of electricity, use of portable electric tools, etc.	
	Chemical	Exposure to substances or preparations, etc.	
	Hygienic	Exposure to physical agents or biological agents, etc.	
2. Personal		Behavior, interaction, psychology, personal trait, capability, etc.	To-Be
3. Job-related	Organization	Management system, work policies, and processes, maintenance of equipment, proper arrangements, etc.	
	Environment	Control of illumination, temperature, humidity, ventilation, pollutants, etc.	
4. System-related		System safety, dangers by others, software integration, cybersecurity, etc.	

## 5 Conclusion

This study discusses the significance of risk assessments in robots. Several countermeasures over some weaknesses of current assessments have been suggested by analyzing three assessment cases and investigating some root causes of operator-injured robot accidents.

Through the analysis and investigation, risk assessments in practice have primarily been focusing on physical aspects despite other hazard factors to identify. On the contrary, operator-injured accidents by robots based on statistical data have been caused by basic reasons such as psychological, organizational perspectives rather than physical aspects. This has led to our conclusion that the scopes be expanded in risk assessments of robots such that other hazard factors besides physical ones are incorporated.

Furthermore, despite the risk assessment, there are remarkable results that 30 to 40 robot accident cases occur every year, 80% of which repetitively take place in similar ways. In this sense, reducing the risks of robot operations and eradicating similar operator-injured accidents by robots are the most challenging issues in the near future for unmanned and autonomous work environments.

For accomplishing these two, it is required that enterprises scrupulously examine the hazards from additional risk perspectives and confirm the reflection of these reasonably foreseeable factors to the risk assessment of robots. These critical factors consider to include personal factors of operators with traits, psychology, and communication; job-related elements with work circumstance, work for climate, organization, and so on; and system-concerned factors, namely software integrity and cybersecurity.

Hereafter, further research on the risk assessment of industrial robots should aim to discover additional hazards about the robots and robotic systems, how to apply the methods of quantifying personal, job-related, and system-concerned hazards, and how to integrate these three hazard factors in more detail with physical factors already assessed. More significantly, the long-term hazards and effects of chronic conditions and injuries in robot workplaces such as hearing loss, skin hypersensitivity, vibration are also one of the areas for future studies on hazard factors of robotic tasks and operators’ injuries.

## Conflicts of interest

The authors declare no conflict of interest.
